# Hematological Changes Associated with Thrombotic Events in Cancer Patients: A Retrospective Exploratory Study

**DOI:** 10.3390/jcm15134998

**Published:** 2026-06-26

**Authors:** Yavuz Katırcılar, İrfan Buğday, Hacer Demir, Mevlüde İnanç

**Affiliations:** 1 Hematology Department, Kayseri City Hospital, Kayseri 38080, Türkiye; yavuzktrclr@hotmail.com; 2 Medical Oncology Department, Kayseri City Hospital, Kayseri 38080, Türkiye; 3 Medical Oncology Department, Afyonkarahisar Health Sciences University, Afyon 03030, Türkiye; 4 Medical Oncology Department, Erciyes University Medical School, Kayseri 38039, Türkiye

**Keywords:** cancer-associated thrombosis, thrombotic events, platelet count, mean platelet volume, platelet distribution width, neutrophil-to-lymphocyte ratio, cancer, thrombosis

## Abstract

**Background**: Cancer-associated thrombosis is a major cause of morbidity and mortality in oncology patients. Routinely available hematological parameters, including platelet count (PLT), mean platelet volume (MPV), platelet distribution width (PDW), and neutrophil-to-lymphocyte ratio (NLR), may reflect thrombo-inflammatory alterations accompanying thrombotic events in malignancy. **Methods**: This retrospective exploratory study included 93 patients with solid malignancies who developed radiologically confirmed thrombotic events between 2006 and 2018. Clinical and laboratory data were retrospectively reviewed. Hematological parameters obtained within seven days before and after thrombotic events were compared using appropriate parametric and non-parametric statistical methods. **Results**: Thrombotic events were most frequently observed in patients with lung, colorectal, breast, and gastric cancers. Gastrointestinal malignancies accounted for 47.3% of cases. Venous thrombotic events represented the majority of cases (63.4%), whereas arterial thrombosis was observed in a smaller subset of patients (10.7%). Pulmonary embolism was identified in 23.7% of patients. Central venous catheter use was significantly associated with subclavian/femoral vein thrombosis (*p* < 0.001). PLT significantly decreased following thrombotic events (329.3 × 10^3^/µL vs. 260.8 × 10^3^/µL, *p* < 0.001), whereas MPV increased modestly (9.23 ± 1.57 fL vs. 9.40 ± 1.45 fL, *p* < 0.001). PDW significantly decreased (14.37 ± 2.78 vs. 13.63 ± 3.24, *p* = 0.011). NLR increased numerically (3.33 ± 2.32 vs. 4.26 ± 4.74) but did not reach statistical significance (*p* = 0.089). An inverse correlation was observed between PLT and MPV (r = −0.268, *p* = 0.009). **Conclusions**: Routinely available hematological parameters, including PLT, MPV, PDW, and NLR, demonstrated measurable alterations in cancer patients with thrombotic events and may reflect thrombo-inflammatory processes associated with malignancy. However, because of the retrospective design, heterogeneous study population, and absence of a non-thrombotic control group, these findings should be considered exploratory and hypothesis-generating rather than evidence of predictive biomarkers. Larger prospective controlled studies are required to clarify their clinical significance.

## 1. Introduction

Venous thromboembolism (VTE), including deep vein thrombosis (DVT) and pulmonary embolism (PE), is one of the most frequent and clinically significant complications in patients with malignancy and remains a major cause of non-cancer-related morbidity and mortality in oncology practice [1,2,3]. Compared with the general population, patients with cancer have a substantially increased risk of thrombotic events because of complex interactions among tumor biology, systemic inflammation, endothelial dysfunction, and activation of coagulation pathways [3,4,5]. In addition to tumor-related mechanisms, cancer-directed therapies such as chemotherapy, radiotherapy, surgery, hospitalization, and the use of central venous catheters may further increase thrombotic risk by promoting vascular injury, platelet activation, and hypercoagulability [5,6,7,8]. Cancer-associated thrombosis therefore represents a major clinical challenge that may adversely affect treatment continuity, quality of life, and survival outcomes.

The pathophysiology of cancer-associated thrombosis is multifactorial and involves dynamic interactions between malignant cells, inflammatory mediators, platelets, leukocytes, and vascular endothelial cells [4,5]. Tumor cells can directly activate coagulation pathways through the expression of tissue factor and cancer procoagulant substances, while inflammatory cytokines released from both tumor and host cells amplify endothelial activation and thrombin generation [5,7]. Increasing evidence also supports the concept of thromboinflammation, emphasizing the close relationship between inflammation and coagulation in malignancy-associated thrombosis [8,9]. These mechanisms collectively create a prothrombotic microenvironment that predisposes cancer patients to both venous and arterial thrombotic complications.

Platelets play a central role not only in primary hemostasis but also in thrombosis, inflammation, angiogenesis, and tumor progression [10,11,12]. Activated platelets contribute to thrombus formation through adhesion, aggregation, and thrombin generation while simultaneously facilitating tumor cell survival and metastatic dissemination through interactions with circulating tumor cells and endothelial surfaces [11,12]. Consequently, platelet-related hematological indices such as platelet count (PLT), mean platelet volume (MPV), and platelet distribution width (PDW) have attracted growing interest as potential markers reflecting platelet activation and thrombotic activity. Increased MPV and altered PDW have been associated with enhanced platelet reactivity and prothrombotic states in several cardiovascular and oncological conditions [13,14,15]. However, currently available findings remain inconsistent, particularly in heterogeneous cancer populations.

Systemic inflammation also plays a pivotal role in cancer progression and thrombosis. Among routinely available inflammatory biomarkers, the neutrophil-to-lymphocyte ratio (NLR) has emerged as a simple and inexpensive parameter reflecting the balance between pro-inflammatory activity and host immune response [16,17]. Elevated NLR has been associated with adverse oncological outcomes in multiple malignancies and has also been linked to thrombotic risk, potentially through cytokine-mediated endothelial dysfunction, neutrophil extracellular trap formation, and dysregulation of coagulation pathways [9,16,17,18]. Nevertheless, the clinical relevance of NLR in the setting of cancer-associated thrombosis remains incompletely understood.

Although several previous studies have investigated platelet indices and inflammatory biomarkers in patients with malignancy, data regarding dynamic hematological changes occurring around thrombotic events remain limited, particularly in real-world oncology populations with heterogeneous tumor types and treatment exposures. Furthermore, most available studies have focused on isolated biomarkers or predictive risk models rather than evaluating temporal changes in routinely available hematological parameters before and after thrombotic events.

Therefore, the present retrospective study was designed as an exploratory analysis to evaluate changes in platelet count (PLT), mean platelet volume (MPV), platelet distribution width (PDW), and neutrophil-to-lymphocyte ratio (NLR) in cancer patients with radiologically confirmed thrombotic events. By examining hematological parameters obtained before and after thrombosis, we aimed to explore whether these routinely accessible markers may reflect thrombo-inflammatory alterations associated with cancer-related thrombosis rather than establish independent predictive biomarkers for thrombotic risk.

## 2. Materials and Methods

### 2.1. Study Design and Patients

This retrospective observational study was conducted at the Medical Oncology Clinic of Health Sciences University Kayseri Training and Research Hospital. The study protocol was approved by the Ethics Committee of Erciyes University Faculty of Medicine (approval number: 2018/293; approval date: 23 May 2018). Institutional permission for retrospective data access was subsequently obtained from Health Sciences University Kayseri Training and Research Hospital.

Medical records of patients diagnosed with solid or hematological malignancies between January 2006 and May 2018 were retrospectively reviewed.

Patients with radiologically confirmed thrombotic events occurring during the course of malignancy were eligible for inclusion. A total of 93 patients with available clinical records and paired laboratory measurements obtained before and after thrombotic events were included in the analysis.

Patients with incomplete laboratory data, active infection, known chronic inflammatory or autoimmune diseases, or missing clinical information were excluded. Because of the retrospective nature of the study, potential confounding effects related to corticosteroid use, anticoagulant treatment, supportive therapies, and variations in chemotherapy regimens could not be fully controlled.

The present study was designed as an exploratory analysis evaluating hematological alterations associated with thrombotic events rather than as a predictive biomarker study. No comparator group of cancer patients without thrombosis was available because of the retrospective design and limitations of accessible institutional data.

### 2.2. Diagnosis of Thrombotic Events

The diagnosis of thrombotic events was confirmed using standard imaging modalities, including Doppler ultrasonography, computed tomography (CT), and angiographic imaging when clinically indicated. Thrombotic events were classified according to anatomical localization and vascular involvement.

Although the primary focus of the study was cancer-associated venous thromboembolism, a limited number of arterial thrombotic events were also included because of retrospective database characteristics and the initial data collection process. Therefore, the findings should be interpreted within the context of a heterogeneous thrombotic population.

### 2.3. Data Collection

Demographic and clinical data were retrieved from institutional electronic medical records. Collected variables included age, sex, primary malignancy type, metastatic status at diagnosis, thrombus localization, smoking history, diabetes mellitus status, central venous catheter use, surgical history, chemotherapy exposure, and radiotherapy history.

Venous thromboembolism was considered chemotherapy-associated when thrombotic events occurred during active systemic anticancer treatment documented in patient records.

Because of incomplete retrospective documentation, detailed information regarding Eastern Cooperative Oncology Group (ECOG) performance status, tumor progression status, thromboprophylaxis, anti-angiogenic therapies, corticosteroid exposure, and timing of anticoagulant initiation was not consistently available for all patients.

### 2.4. Laboratory Parameters

Complete blood count parameters were obtained from routine laboratory tests performed during standard clinical follow-up. Evaluated hematological parameters included platelet count (PLT), mean platelet volume (MPV), platelet distribution width (PDW), neutrophil count, lymphocyte count, and neutrophil-to-lymphocyte ratio (NLR).

NLR was calculated by dividing the absolute neutrophil count by the absolute lymphocyte count.

To minimize temporal variability, laboratory measurements obtained within seven days before and seven days after thrombotic event diagnosis were recorded and compared. Because of retrospective data limitations, it could not be reliably determined whether post-thrombotic laboratory measurements were obtained before or after initiation of anticoagulant therapy.

### 2.5. Statistical Analysis

Statistical analyses were performed using SPSS software version 21.0 (IBM Corp., Armonk, NY, USA). Normality of continuous variables was evaluated using the Shapiro–Wilk test.

Continuous variables were expressed as mean ± standard deviation, median (minimum–maximum), or frequencies and percentages where appropriate. Paired sample *t*-tests were used for normally distributed paired comparisons, whereas the Wilcoxon signed-rank test was applied for non-normally distributed variables.

Correlation analyses between hematological parameters were performed using Pearson or Spearman correlation coefficients as appropriate. In addition to *p*-values, 95% confidence intervals were calculated for principal paired comparisons to facilitate interpretation of effect magnitude and clinical relevance.

Multivariable regression analysis was not performed because of the limited sample size, heterogeneous study population, and incomplete availability of several clinically relevant covariates.

Given the exploratory design, limited sample size, and hypothesis-generating nature of the study, formal correction for multiple comparisons was not applied; therefore, statistically significant findings should be interpreted cautiously.

A two-sided *p*-value < 0.05 was considered statistically significant.

### 2.6. Ethical Considerations

The study protocol was initially approved by the Ethics Committee of Erciyes University Faculty of Medicine, and institutional permission for retrospective data access was subsequently obtained from Kayseri Training and Research Hospital (approval number: 2018/293; approval date: 23 May 2018). The study was conducted in accordance with the principles of the Declaration of Helsinki.

Given the retrospective design and anonymized data collection process, the requirement for informed consent was waived by the ethics committee.

## 3. Results

### 3.1. Patient Characteristics

A total of 93 patients with malignancy-associated thrombotic events were included in the study. Of these patients, 48 (51.6%) were male and 45 (48.4%) were female. The median age of the cohort was 62 years (range: 34–82 years). The distribution of primary malignancies is summarized in Table 1.

### 3.2. Cancer Types

The distribution of primary malignancies is presented in Table 1. Thrombotic events were most frequently observed in patients with lung cancer (20.4%), colorectal cancer (19.4%), breast cancer (16.1%), and gastric cancer (14.0%). When grouped according to organ systems, gastrointestinal malignancies accounted for 47.3% of all cases and represented the largest subgroup in the cohort.

### 3.3. Localization of Thrombotic Events

The anatomical distribution of thrombotic events is summarized in Table 2.

Venous thromboembolism accounted for the majority of cases (63.4%), whereas arterial thrombosis was observed in 10.7% of patients. Pulmonary embolism was present in 23.7% of patients. No statistically significant association was observed between cancer type and thrombus localization (*p* = 0.180).

### 3.4. Clinical Risk Factors

Among the study population, 40 patients (43.0%) were aged ≥ 65 years. Diabetes mellitus was present in 15.1% of patients, while 45.2% were active smokers. Smoking status was not significantly associated with thrombotic event localization or distribution (*p* = 0.750).

Central venous catheters were present in nine patients (9.7%). Of these patients, five (55.6%) developed thrombosis involving the subclavian or femoral veins. This association was statistically significant (*p* < 0.001).

### 3.5. Hematological Parameters

**Figure 1:**
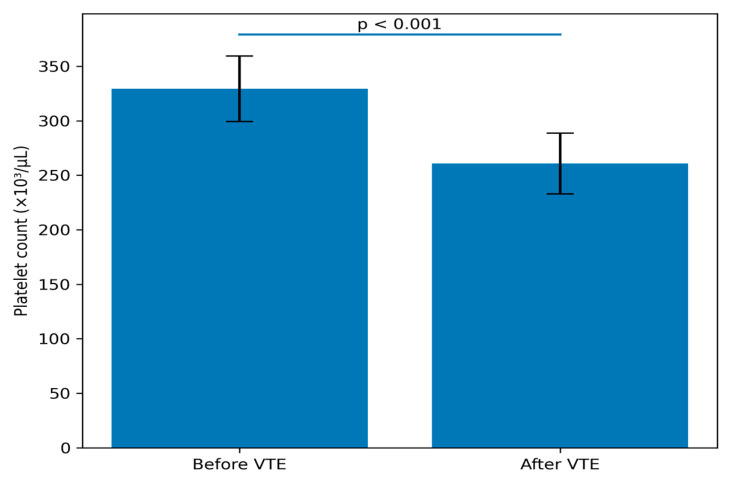
Changes in Platelet Count Before and After VTE.

**Figure 2:**
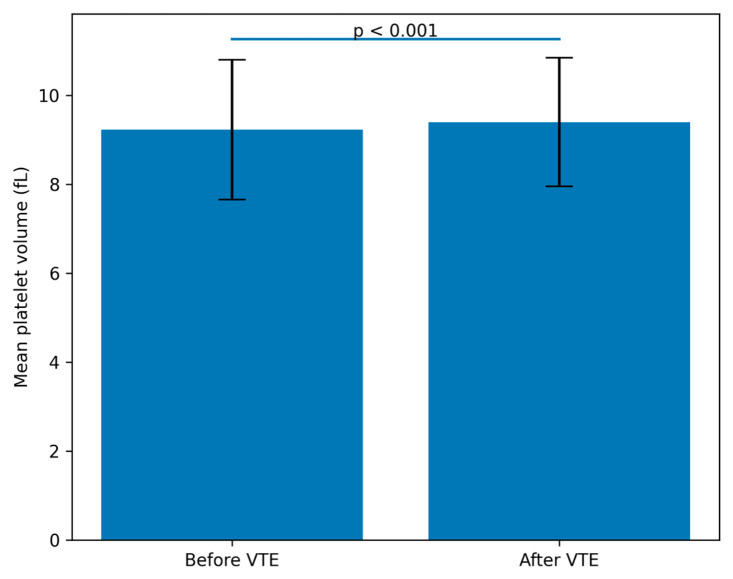
Changes in Mean Platelet Volume Before and After VTE.

Changes in hematological parameters before and after thrombotic events are presented in Table 3 and illustrated in Figure 1 and Figure 2.

Mean platelet count (PLT) significantly decreased following thrombotic events, from 329.3 × 10^3^/µL before thrombosis to 260.8 × 10^3^/µL after thrombosis (*p* < 0.001). Mean platelet volume (MPV) increased modestly from 9.23 ± 1.57 fL to 9.40 ± 1.45 fL (*p* < 0.001). Platelet distribution width (PDW) significantly decreased from 14.37 ± 2.78 to 13.63 ± 3.24 (*p* = 0.011).

Neutrophil-to-lymphocyte ratio (NLR) increased numerically from 3.33 ± 2.32 to 4.26 ± 4.74; however, the difference did not reach statistical significance (*p* = 0.089).

Detailed descriptive statistics, including mean differences and 95% confidence intervals for paired comparisons, are presented in Table 3.

Correlation analysis demonstrated a statistically significant inverse relationship between PLT and MPV (r = −0.268, *p* = 0.009).

### 3.6. Treatment-Related Findings

Thrombotic events occurred during active chemotherapy in 58 patients (62.4%). Among the total cohort, five patients (5.4%) developed thrombotic events within the first three months after chemotherapy initiation, whereas one patient (1.1%) developed thrombosis between three and six months. Detailed treatment-timing information was unavailable for the remaining patients because of retrospective data limitations.

Additionally, thrombotic events occurred during radiotherapy in four patients (4.3%) and following surgical intervention in five patients (5.4%).

## 4. Discussion

Venous thromboembolism remains one of the most clinically significant complications in patients with malignancy and continues to contribute substantially to morbidity and mortality in oncology practice [1,2,3,4]. In the present retrospective exploratory study, we evaluated dynamic changes in routinely available hematological parameters in cancer patients before and after thrombotic events. The principal findings of this study demonstrate that platelet count (PLT), mean platelet volume (MPV), and platelet distribution width (PDW) showed measurable alterations in association with thrombotic events, whereas neutrophil-to-lymphocyte ratio (NLR) increased numerically without reaching statistical significance.

Cancer-associated thrombosis is a multifactorial process driven by complex interactions among tumor biology, systemic inflammation, endothelial injury, platelet activation, and treatment-related factors such as chemotherapy and central venous catheter use [2,3,4,5]. In agreement with previous reports, lung, gastrointestinal, and breast malignancies represented the most frequent cancer types in our cohort, supporting the established observation that these malignancies are associated with an increased thrombotic burden [3,4,5,19,20,21,22,23,24]. Similarly, the significant association observed between central venous catheter use and upper extremity thrombosis is consistent with prior studies demonstrating the contribution of catheter-related endothelial injury and altered venous flow to thrombus formation [6,7].

Platelets are increasingly recognized as key mediators linking thrombosis, inflammation, and cancer progression [8,9,10,11,12]. Beyond their traditional hemostatic role, activated platelets may facilitate thrombin generation, endothelial adhesion, angiogenesis, immune evasion, and metastatic dissemination of tumor cells [10,11,12]. In the present study, PLT significantly decreased following thrombotic events, whereas MPV demonstrated a modest but statistically significant increase. In addition, an inverse correlation was observed between PLT and MPV. These findings may reflect increased platelet activation and consumption during acute thrombotic processes. Larger platelets are considered metabolically and enzymatically more active and may exhibit enhanced prothrombotic potential through increased granule content and thromboxane A2 production [13,14,15].

Similar findings have been reported in previous studies evaluating platelet volume indices in thrombotic and cardiovascular disorders. Gasparyan et al. suggested that increased MPV may reflect enhanced platelet activation and a prothrombotic state, whereas Vizioli et al. reported associations between MPV and adverse cardiovascular outcomes [14,15]. Nevertheless, the clinical utility of MPV remains controversial because reported effect sizes are generally modest and findings have not been consistent across different patient populations. Consistent with these observations, the absolute magnitude of MPV change observed in our cohort was relatively small despite statistical significance. Therefore, the clinical relevance of this difference should be interpreted cautiously. Consequently, although MPV and related platelet indices may reflect thrombo-inflammatory alterations, the present findings do not support their use as independent predictive biomarkers for thrombotic risk.

PDW, which reflects variability in platelet size distribution, also demonstrated a significant decrease after thrombotic events. Previous studies evaluating PDW in thrombotic and cardiovascular disorders have reported inconsistent findings. While some investigators suggested that altered PDW may reflect increased platelet activation and thrombotic tendency, others failed to demonstrate a consistent association with thrombotic outcomes. Therefore, the biological significance of the observed decrease in PDW remains uncertain and should be interpreted cautiously. Further prospective studies are needed to clarify its potential role in cancer-associated thrombosis.

Systemic inflammation represents another important component of cancer-associated thrombosis. NLR has emerged as an accessible inflammatory biomarker associated with poor oncological outcomes and adverse systemic inflammatory states in several malignancies [16,17,18]. In our study, NLR increased numerically following thrombotic events but did not reach statistical significance. This finding may be related to the relatively small sample size; heterogeneity of cancer types; variability in treatment exposures; and the influence of uncontrolled confounding factors such as infection, corticosteroid use, and chemotherapy-related inflammatory changes.

Recent evidence from hematological disorders has further highlighted the importance of inflammatory pathways and leukocyte-derived biomarkers. Stefaniuk et al. demonstrated that NLR and lymphocyte-to-monocyte ratio may provide prognostic information across various hematological malignancies [25]. In addition, Barbui and Scandura emphasized the role of inflammatory mechanisms in myeloproliferative neoplasms and proposed novel therapeutic end points targeting both clonal and inflammatory pathways [26]. More recently, Cavalca et al. reported that elevated NLR at diagnosis independently predicted venous thrombosis in patients with prefibrotic primary myelofibrosis [27]. These findings support the concept that inflammation and thrombosis are closely interconnected and suggest that leukocyte-derived biomarkers may contribute to thrombotic risk stratification in selected hematological conditions. However, because our cohort consisted predominantly of patients with heterogeneous solid tumors rather than hematological neoplasms, direct comparisons should be interpreted with caution.

Therefore, NLR should be interpreted as a nonspecific inflammatory marker rather than a thrombosis-specific indicator in the present clinical setting. Although accumulating evidence supports the role of inflammatory pathways and suggests a potential contribution of NLR to thromboinflammatory processes, larger prospective studies are required to determine whether NLR provides clinically meaningful information beyond established thrombosis risk factors and prediction models [16,17,18,25,26,27].

One of the most important limitations affecting interpretation of the present findings is the absence of a comparator group of cancer patients without thrombotic events. Without a matched non-thrombotic oncology cohort, it is not possible to determine whether the observed hematological alterations are directly related to thrombosis or instead reflect underlying malignancy, systemic inflammation, or treatment-related effects. Furthermore, because laboratory measurements obtained after thrombosis may have been influenced by anticoagulant therapy, hospitalization, or acute inflammatory responses, causal interpretation remains limited.

From a clinical perspective, routinely available hematological parameters such as PLT, MPV, PDW, and NLR may provide supportive information regarding thrombo-inflammatory changes in patients with cancer. These markers are inexpensive, rapidly obtainable, and widely integrated into routine complete blood count analyses. Nevertheless, given the retrospective design, heterogeneous patient population, lack of multivariable adjustment, and absence of prospective validation, their independent predictive value cannot be established based on the present findings. Future prospective controlled studies incorporating standardized oncological and thrombotic risk assessment tools, including the Khorana score, are required to determine whether these hematological parameters provide clinically meaningful incremental value beyond established thrombosis risk models.

Despite these limitations, the present study provides real-world data regarding dynamic hematological changes occurring around thrombotic events in oncology patients. The paired comparison design allowed assessment of temporal alterations in routinely available laboratory parameters within the same patient population, thereby reducing interindividual variability to some extent. These findings may contribute to the growing body of evidence exploring the relationship between thromboinflammation and malignancy-associated thrombosis.

## 5. Conclusions

In conclusion, this study demonstrated that routinely available hematological parameters, particularly platelet count (PLT), mean platelet volume (MPV), and platelet distribution width (PDW), exhibited measurable but modest alterations in cancer patients following thrombotic events, whereas no significant change was observed in the neutrophil-to-lymphocyte ratio (NLR). These findings suggest that routine hematological indices may reflect thrombo-inflammatory changes accompanying cancer-associated thrombosis.

However, because of the retrospective design, heterogeneous patient population, absence of a non-thrombotic control group, and potential confounding factors, no conclusions regarding causality or independent predictive value can be drawn. Therefore, the present findings should be considered exploratory and hypothesis-generating.

Larger prospective multicenter studies incorporating appropriate control groups and multivariable analyses are required to clarify the clinical significance and potential clinical utility of these hematological alterations in cancer-associated thrombosis.

## Figures and Tables

**Table 1:** Distribution of patients according to primary cancer diagnosis (n = 93).

Cancer System/Type	n	%
**Gastrointestinal malignancies**		
Colon cancer	18	19.4
Rectal cancer	6	6.5
Stomach cancer	13	14.0
Pancreatic cancer	4	4.3
Esophageal cancer	1	1.1
Hepatocellular carcinoma	2	2.2
**Subtotal (GI)**	**44**	**47.3**
**Thoracic malignancies**		
Lung cancer	19	20.4
Thymoma	1	1.1
**Subtotal (Thoracic)**	**20**	**21.5**
**Breast malignancy**		
Breast cancer	15	16.1
**Subtotal (Breast)**	**15**	**16.1**
**Genitourinary malignancies**		
Renal cell carcinoma	2	2.2
Prostate cancer	3	3.2
Endometrial cancer	4	4.3
**Subtotal (GU)**	**9**	**9.7**
**Other malignancies**		
Thyroid cancer	1	1.1
Melanoma	1	1.1
Leiomyosarcoma	1	1.1
Testicular tumor	1	1.1
**Subtotal (Other)**	**4**	**4.3**
**Total**	**93**	**100**

**Table 2:** Distribution of thrombotic event locations (n = 93).

Thrombotic System	Location	n	%
**Venous thromboembolism**			
	Superficial femoral vein	16	17.2
	Deep femoral vein	12	12.9
	Popliteal vein	7	7.5
	Superficial + deep femoral vein	7	7.5
	Jugular vein	2	2.2
	Subclavian vein	6	6.5
	Cephalic vein (upper extremity)	7	7.5
	Renal vein	2	2.2
	**Portal vein**	**2**	**2.2**
	**Subtotal (venous)**	**61**	**65.6**
**Arterial thrombosis**			
	Femoral artery	1	1.1
	Iliac artery	3	3.2
	Brachial artery	2	2.2
	Cerebral artery	2	2.2
	Aorta	2	2.2
	**Subtotal (arterial)**	**10**	**10.7**
Pulmonary involvement *			
	Pulmonary embolism	22	23.7
	**Subtotal (pulmonary)**	**22**	**23.7**
	**Total**	**93**	**100**

* Pulmonary embolism cases were analyzed separately from peripheral venous thrombosis because pulmonary involvement may occur with or without documented deep venous thrombosis.

**Table 3:** Comparison of hematological parameters before and after thrombotic events in cancer patients (n = 93).

Parameter	Pre	Post	Mean Difference (Pre − Post)	95% CI	*p*
**PLT**	329.28 ± 140.75	260.76 ± 134.29	−68.51	−101.88 to −35.15	<0.001
**PDW**	14.37 ± 2.78	13.63 ± 3.24	−0.74	−1.31 to −0.18	0.011
**MPV**	9.23 ± 1.57	9.40 ± 1.45	−0.52	−0.79 to −0.24	<0.001
**NLR**	3.33 ± 2.32	4.26 ± 4.74	−0.93	−2.002 to 0.145	0.089

Abbreviations: PLT, platelet count; MPV, mean platelet volume; PDW, platelet distribution width; NLR, neutrophil-to-lymphocyte ratio; CI, confidence interval.

## Data Availability

No new data were created or analyzed in this study.
